# Interaction of Camptothecin Anticancer Drugs with Ribosomal Proteins L15 and L11: A Molecular Docking Study

**DOI:** 10.3390/molecules28041828

**Published:** 2023-02-15

**Authors:** Christian Bailly, Gérard Vergoten

**Affiliations:** 1 Institut de Chimie Pharmaceutique Albert Lespagnol (ICPAL), Faculté de Pharmacie, University of Lille, 3 rue du Professeur Laguesse, BP-83, F-59006 Lille, France; 2 CNRS, Inserm, CHU Lille, UMR9020-U1277—CANTHER—Cancer Heterogeneity Plasticity and Resistance to Therapies, University of Lille, F-59000 Lille, France; 3 OncoWitan, Consulting Scientific Office, Wasquehal, F-59290 Lille, France

**Keywords:** anticancer agents, belotecan, camptothecin, molecular docking, ribosomal protein, topoisomerase I, topotecan

## Abstract

The antitumor drug topotecan (TPT) is a potent inhibitor of topoisomerase I, triggering DNA breaks lethal for proliferating cancer cells. The mechanism is common to camptothecins SN38 (the active metabolite of irinotecan) and belotecan (BLT). Recently, TPT was shown to bind the ribosomal protein L15, inducing an antitumor immune activation independent of topoisomerase I. We have modeled the interaction of four camptothecins with RPL15 derived from the 80S human ribosome. Two potential drug-binding sites were identified at Ile135 and Phe129. SN38 can form robust RPL15 complexes at both sites, whereas BLT essentially gave stable complexes with site Ile135. The empirical energy of interaction (ΔE) for SN38 binding to RPL15 is similar to that determined for TPT binding to the topoisomerase I-DNA complex. Molecular models with the ribosomal protein L11 sensitive to topoisomerase inhibitors show that SN38 can form a robust complex at a single site (Cys25), much more stable than those with TPT and BLT. The main camptothecin structural elements implicated in the ribosomal protein interaction are the lactone moiety, the aromatic system and the 10-hydroxyl group. The study provides guidance to the design of modulators of ribosomal proteins L11 and L15, both considered anticancer targets.

## 1. Introduction

**Figure 1:**
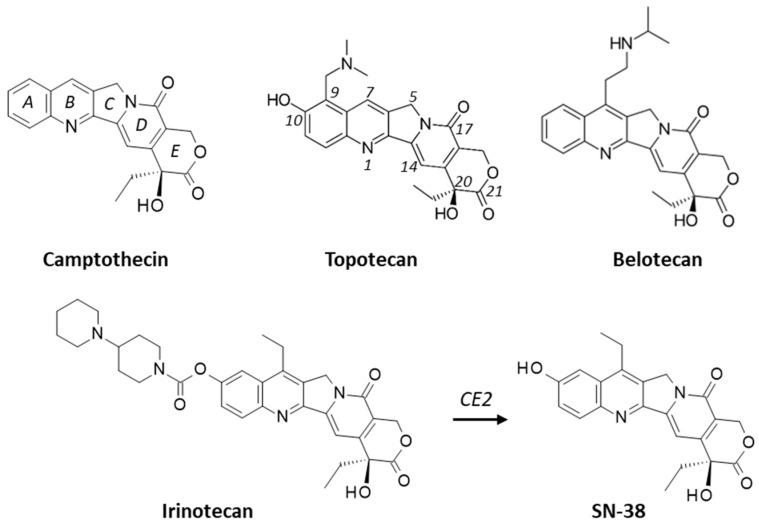
Structure of camptothecin (CPT) and its derivatives, topotecan (TPT), belotecan (BTC) and irinotecan (IRT). IRT is a prodrug, activated upon release of the active metabolite SN-38 after cleavage of the ester function by the enzyme carboxylesterase 2 (CE2). The numbering scheme is indicated for CPT (rings A–E) and TPT (atom numbering positions).

Camptothecin (CPT) derivatives play an important role in the treatment of cancers, solid tumors in particular. Currently, there are five CPT-based approved anticancer drugs. The first one is irinotecan (IRT, Camptosar^®^, first approved in 1994), mainly used to treat colon, gastric and pancreatic cancers, in combination with other cytotoxic drugs, targeted therapeutics or immunotherapy [1,2]. The IRT active metabolite SN38 (Figure 1) functions as a potent inhibitor of the DNA-manipulating enzyme topoisomerase I, via stabilization of the cleavable DNA–protein complex. The drug-induced DNA breaks are lethal if they are not repaired [3]. The second one is topotecan (Hycamtin^®^, FDA-approved in 1996), mainly used to treat ovarian cancer and in the second-line setting to treat relapsed small-cell lung cancer (SCLC) [4]. In patients with resistant/refractory SCL tumors, topotecan is the only agent able to increase overall survival compared with the best supportive care [5]. The third drug is belotecan (Camtobell^®^, 2003), only approved in South Korea for the treatment of ovarian cancer and SCLC. The fourth drug, nal-IRI (Onivyde^®^), corresponds to a nanoliposomal formulation of IRT, approved in 2015 for the treatment of advanced pancreatic cancer [6,7]. The last drug is the antibody–drug conjugate (ADC) sacituzumab govitecan (SG), which combines an anti-TROP-2 (trophoblast cell-surface antigen 2) antibody coupled to SN-38. This ADC is used to treat patients with metastatic triple-negative breast cancer and those with locally advanced and metastatic urothelial cancer [8,9,10]. These five drugs target topoisomerase I, a four-decade-old target that remains an attractive protein for drug design. In each case, the CPT drug binds at the site of DNA cleavage by intercalating between base pairs, thus preventing religation of the cleaved strand [11]. Novel topoisomerase I inhibitors remain searched, including both CPT-based products and new scaffolds acting as topoisomerase I “poisons” stabilizing the enzyme-mediated DNA cleavage complex [12,13]. Nobody disputes the fact that topoisomerase I is a major anticancer target and the main molecular target of CPT and its many derivatives, including SN-38 and topotecan.

Besides topoisomerase I, topotecan and SN38 can interact with other proteins implicated in drug transport or partly responsible for the drug resistance mechanisms or tumor cell killing. This is the case for the multidrug transporter ABCG2, which can accommodate a topotecan molecule in a binding pocket, affecting the drug’s pharmacokinetic properties and contributing to the resistance of cancer cells to TPT [14,15]. TPT can interact also with other drug transporters, such as the multidrug resistance protein 1 (MRP1) [16]. Another CPT-binding protein is toll-like receptor 4 (TLR4), implicated in intestinal damage and late-onset diarrhea induced by IRT treatment [17]. SN38 has been shown to affect TLR4 via binding to the TLR4/MD-2 complex, in which dimerization is necessary to trigger the production of proinflammatory cytokines and interferon. Apparently, both the lactone (closed) form and the carboxylate (open) form of SN38 can interact with the MD-2 molecule, according to a molecular docking analysis [18,19]. Other potential protein targets for TPT have been proposed, such as death-associated protein kinase 1 (DAPK1) [20]. In addition, CPT and SN-38 have been shown to inhibit the binding of the transcriptional regulator protein FUBP1 (FUSE binding protein 1) to its single-stranded target DNA FUSE, possibly via direct targeting of the protein [21]. FUBP1 plays a role in DNA repair, and its blockade with SN38 can enhance DNA damage and promote the killing of cancer cells [22]. The p53-binding protein MDM2 (mouse double minute 2 homolog) is another potential target protein for IRT. MDM2 is an E3 ubiquitin ligase that targets the tumor suppressor p53 for proteasomal degradation. IRT can bind to both MDM2 and to the antiapoptotic protein Bcl-xL, a member of the Bcl-2 family. The dual targeting of MDM2 and Bcl-xL can facilitate the drug’s anticancer action [23]. A topoisomerase I-independent mechanism has been evoked also to explain the capacity of TPT to decrease the replication of human immunodeficiency virus type 1 (HIV-1) [24]. Finally, a deep learning methodology designated deepDTnet has revealed that TPT can selectively target human retinoic-acid-receptor-related orphan receptor-gamma t (ROR-γt), acting as a receptor antagonist and as such potentially useful for the treatment of multiple sclerosis [25]. In other words, SN38 and TPT are potent topoisomerase I inhibitors, but the direct modulation of other cellular proteins is not excluded.

A recent study has pointed out the capacity of TPT to target the 60S ribosomal protein RPL15, inhibiting preribosomal subunit formation, so as to induce antitumor immune activation independent of topoisomerase I [26]. The binding of TPT to RPL15 inhibits its interaction with the partner protein RPL4s, so as to decrease RPL4 stability and then to activate an immune response through the secretion of DAMPs (damage-associated molecular patterns) [26]. The drug is a potent DAMP inducer, capable of triggering dendritic cell activation and cytokine production [27]. These effects can result directly from the binding of TPT to the 60S ribosomal protein RPL15 [28]. But how does TPT bind to RPL15? To which protein site? What are the drug elements implicated in the interaction? These remain open questions that we have addressed using a molecular modeling approach. We have modeled the interaction between four camptothecin compounds (CPT, SN38 TPT and BTC (Figure 1)) to the protein RPL15, which is a component of the 80S human ribosome. In addition, we built models of the same compounds interacting with the analogous protein RPL11, which is known to be sensitive to various topoisomerase inhibitors, including topotecan [29]. From the different protein–drug models, binding energies have been compared, and structure–binding relationships have been defined.

## 2. Results

### 2.1. Interaction of Camptothecins with RPL15

**Figure 2:**
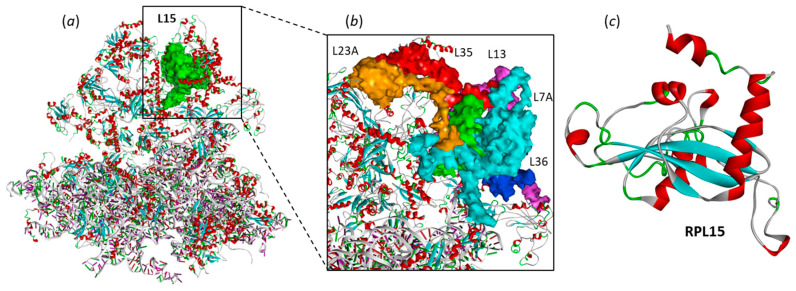
The ribosomal protein L15 (RPL15). (**a**) RPL15 (in green) within the human 80S ribosome (from PDB: 4UG0). (**b**) A view of RLP15 and the five surrounding ribosomal proteins L7A, L13, L23A, L35 and L36. (**c**) Molecular model of RPL15 isolated from the ribonucleoprotein complex, with α-helices (in red) and β-sheets (in cyan).

We started our investigation using the structure of the human 80S ribosome, a large ribonucleoprotein complex with multiple ribosomal RNA and protein entities (PDB: 4UG0). This structure is an essential component of the translational machinery that catalyzes protein synthesis [30]. The structure of RPL15 was extracted from the complex, together with that of the surrounding proteins, those directly in contact with RPL15, proteins L7A, L13, L23A, L35 and L36, as represented in Figure 2. These five proteins interact with RPL15 but do not completely shield the protein, which remains accessible from different positions. This subanalysis allows defining the regions of L15 free of access. A drug docking analysis was then performed with L15 alone, prior to replacing the different binding poses obtained in the global protein environment.

**Figure 3:**
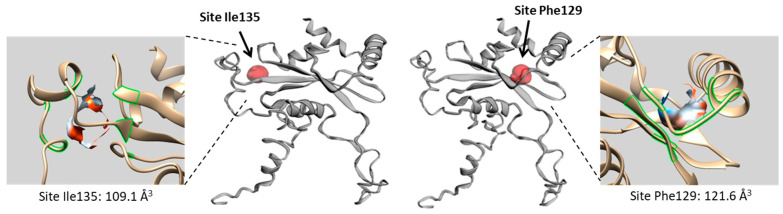
Binding site analysis of RPL15 using web server CASTp 3.0 revealed two potential sites located around residues Ile135 and Phe129, on each side of the β-sheet plane, as shown (red area). In both cases, a detailed view of the binding site is shown with the contact surface delimited in green and the hydrophobicity area colored. Site Phe129 has a surface of 131.1 Å^2^ and a volume of 121.6 Å^3^. Site Ile135 presents a surface of 117.1 Å^2^ and a volume of 109.1 Å^3^.

From a structural viewpoint, L15 is a small and compact protein (204 amino acids) with a central β-sheet floor and adjacent helicoidal fragments (Figure 2c). Potential binding sites for camptothecin (CPT) and its two derivatives SN38 and TPT were searched using the web server CASTp 3.0, which is a convenient tool to predict the position of drug-binding sites [31]. Two potential sites emerged from the CAST analysis, located around residues Ile135 and Phe129, as represented in Figure 3. The two sites, located on each side of the β-sheet floor, are equivalent, but Phe129 seems to be more accessible than the Ile135 site. The solvent-accessible surfaces at each site have been determined, according to a standard method [32]. Site Phe129 presents a larger volume (121.6 Å^3^) compared to site Ile135 (109.1 Å^3^). The molecular surface envelope of the former site offers better opportunities for drug binding.

**Figure 4:**
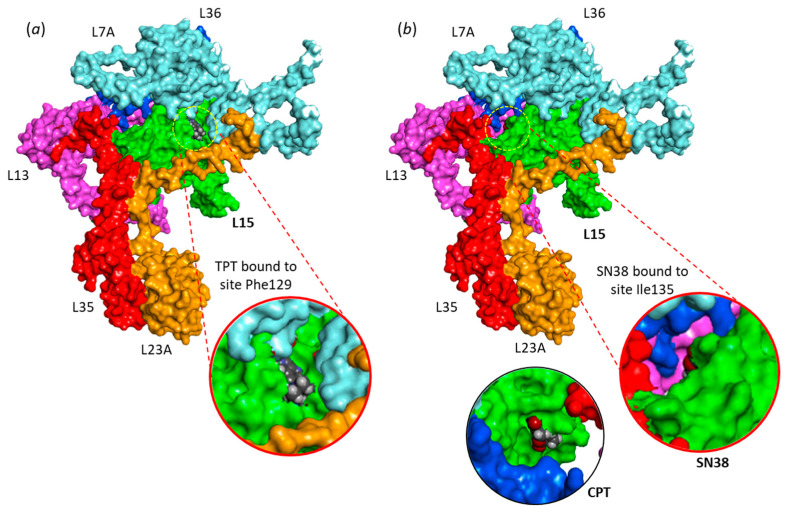
Drug binding to RPL15. (**a**) Model of TPT bound to site Phe129. The drug is inserted into a deep cavity (close-up view). (**b**) Model of SN38 bound to site Ile135. In this case, the SN38 molecule is almost completely buried in the binding site (close-up view). In contrast, at the same site, CPT does not enter well into the site. A large portion of the CPT molecule (the lactone moiety) remains outside the cavity, as shown in the detailed view.

**Figure 5:**
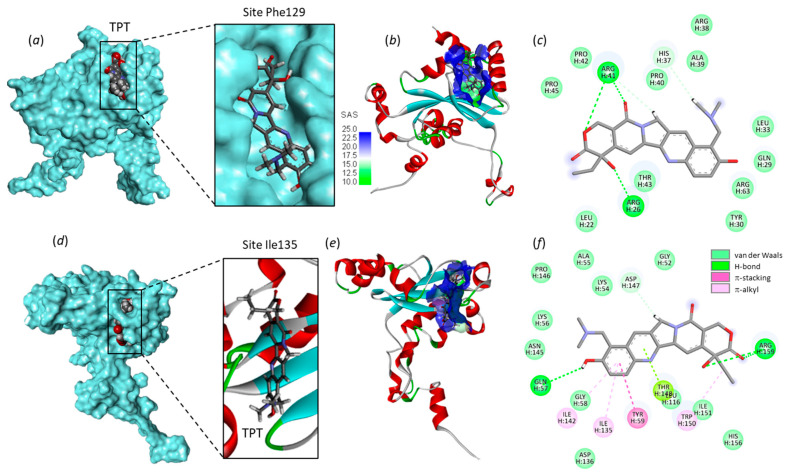
Models of TPT binding to RPL15. The top part (**a**–**c**) shows TPT bound to site Phe129, with (**a**) the TPT molecule inserted into a groove around Phe129 and (**b**) a detailed view of TPT inserted into the binding cavity, with the solvent-accessible surface (SAS) surrounding the drug-binding zone (color code indicated). (**c**) Binding map contacts for TPT bound to the Phe129 site. The bottom part (**d**–**f**) shows TPT bound to site Ile135, with (**d**) the drug extended into the cavity and (**e**) a close-up view of the binding area and the solvent-accessible surface (SAS). (**f**) Binding map contacts for TPT bound to the Ile135 site (color code indicated).

Drug binding to each site was analyzed. The empirical energy of interaction (ΔE) and energy of hydration (ΔG) were calculated and compared for the different products (Table 1). The calculated energies are roughly equivalent at the two sites for the three compounds. The best ligand appears to be SN-38, followed by TPT, and then CPT, which turns out to be a relatively poor ligand of RPL15. SN38 is by far the best compound in terms of binding to the protein. This metabolite of irinotecan can form stable complexes with RPL15 at the Phe129 site and/or the Ile135 site, with binding to the former site being slightly favored. Models of TPT bound to Phe129 and SN38 bound to Ile135 are presented in Figure 4. SN38 inserts deeply into the site; the drug is almost completely buried into the binding cavity, with a tiny portion remaining accessible. In contrast, CPT does not insert well into the same cavity, and the majority of the lactone ring remains out of the binding cavity, as illustrated in Figure 4b. A more detailed view of TPT binding to RPL15 is shown in Figure 5, with the ligand positioned at each site. There is clearly a short but deep groove at Phe129, offering a cavity for TPT binding. The drug sits on the floor of the β-sheet and orients its lactone unit toward Arg26 and Arg41, both implicated in H-bond interactions with the drug (Figure 5a). On the other side of the β-sheet floor, the Ile135 site offers a wider cavity, fully accessible to the solvent, in which the TPT molecule can sit. In this case, the drug interacts with Arg159 and Gln57 (Figure 5b). Similar models have been obtained with SN-38 (not shown). The Phe129 site is smaller and deeper than the Ile135 site, which is more open and susceptible to accommodate bulkier molecules. This is exactly what we observed (*vide infra*).

**Figure 6:**
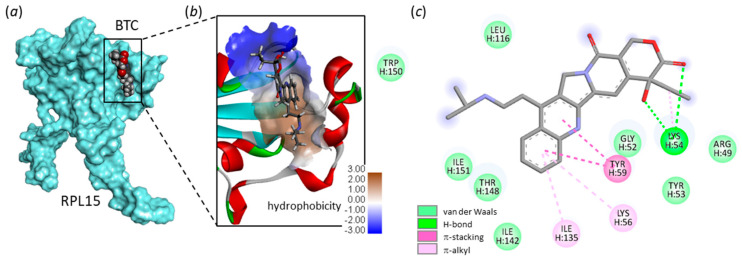
Binding model for belotecan (BTC) bound to RPL-15. (**a**) BTC bound to site Ile135. (**b**) A detailed view of BTC inserted into the binding cavity, with the hydrophobicity area at the drug-binding zone (color code indicated). (**c**) Binding map contacts for BTC bound to site Ile135 (color code indicated).

SN38 and TPT only differ by the nature of the substituent on the A- or B-ring: an ethyl group on the B-ring for SN38 versus a dimethylaminomethyl group on the A-ring for TPT. The favored binding of SN38 to RPL15 compared to TPT suggested that substitution on the B-ring could be important (or at least less detrimental than the A-ring substitution). To investigate this point, we then tested the Korean drug belotecan (BTC), which possesses a slightly longer side chain on the B-ring compared to TPT (Figure 1). Interestingly, we observed that belotecan can bind very well to the Ile135 site (ΔE = −80.4 kcal/mol), with a relative affinity comparable to that of SN38 (ΔE = −79.3 kcal/mol), but its binding to the other site, Phe129, is less favorable (ΔE = −63.3 and −83.7 kcal/mol, for belotecan and SN38, respectively). The drug is certainly too long or too bulky to fit properly into the short Phe129 site, but it can adapt easily to the wider Ile135 site, as represented in Figure 6.

The drug–protein contacts are very similar for SN38 and belotecan. In both cases, the drug engages its lactone carbonyl group into an H-bond with residue Lys54 and a π–π stacking interaction with residue Tyr59. In fact, the isopropylaminoethyl side chain on the B-ring of belotecan does not contribute to the protein interaction. The stability of the belotecan-RPL15 complex is maintained by a set of van der Waals contacts and π-alkyl interactions, in addition to the above-mentioned contacts. The key elements of the drug–protein complexes are the lactone ring and the aromatic chromophore, which allows a stacking interaction with a key tyrosine residue of the protein, not the nature of the alkyl side chain on the A- or B-ring. The hydroxyl group on the A-ring of TPT and SN-38 is a positive element for binding. In both cases, this 10-OH group is implicated in an H-bond with Gly58 (SN-38) or Gln57 (TPT) (Figure 5).

The first part of the docking analysis suggests that (i) the camptothecin drugs can form stable complexes with RPL15, (ii) two potential sites have been identified, around residues Phe129 and Ile135, (iii) binding of SN-38 to site Phe129 represents the most favorable option, and (iv) three drug elements play an important role in the protein interaction: the lactone ring, the aromatic core and the A-ring 10-OH group common to TPT and SN-38.

### 2.2. Drug Binding to RPL15 versus Topoisomerase I and ABCG2

We compared the binding energies calculated with SN-38 and TPT interacting with RPL15, with the energies calculated using the same modeling process with the known targets for the drug, which are the topoisomerase I-DNA complex and the drug transporter ABCG2. The Protein Data Bank provides three structures: (1) CPT bound to a topoisomerase I-DNA complex, with the drug intercalated between two adjacent DNA-base pairs at the enzyme cleavage site (PDB: 1TI8); (2) a similar complex with TPT interfacing with the topoisomerase I-DNA complex, with the drug also intercalated at the cleavage site (PDB: 1K4T); and (3) the structure of TPT bound to the drug transporter ABCG2 (PDB: 7NEZ). The empirical energies of interaction (ΔE) were calculated and compared (Table 2).

The most favorable situation was observed with CPT bound to the topoisomerase I-DNA complex (1TI8). In this case, the calculated ΔE value was −114.20 kcal/mol. Then comes the model of TPT bound to topoisomerase I-DNA complex (1K4T), with a ΔE value of −80.10 kcal/mol. This value is very similar to that obtained for the binding of SN-38 to the RPL15 protein, be it the Ile135 site (ΔE = −79.3 kcal/mol) or the Phe129 site (ΔE = −83.7 kcal/mol). In other terms, the affinity of SN-38 for RPL15 is comparable to that of TPT for the topoisomerase I-DNA complex. Binding of TPT to the ABCG2 transporter (7NEZ) afforded a weak binding energy (ΔE) of −44.55 kcal/mol). The comparison is important because it suggests that TPT presents a higher affinity for the topoisomerase I-DNA complex compared to RPL15, but the binding of TPT to the ribosomal protein is very significant, much better than binding to ABCG2 and comparable to the affinity of the irinotecan metabolite SN38 for the main target, the topoisomerase I-DNA complex.

### 2.3. Interaction of Camptothecins with RPL11

Next, we extended our investigation using the human ribosomal protein L11 for which there is a high-resolution (2.40 Å) X-ray structure available (PDB: 4XXB). It derives from the structure of the binary complex between RPL11 and ubiquitin ligase protein MDM2 (mouse double minute 2 homolog), which is a key suppressor factor for the tumor suppressor gene p53 [33]. RPL11 is a small ribosomal protein of 178 amino acids. Importantly, it has been shown recently that an RPL11-mediated nucleolar stress response regulates the sensitivity of cancer cells to topoisomerase inhibitors, including topotecan [29]. The information prompted us to analyze the potential binding of camptothecins to RPL11.

**Figure 7:**
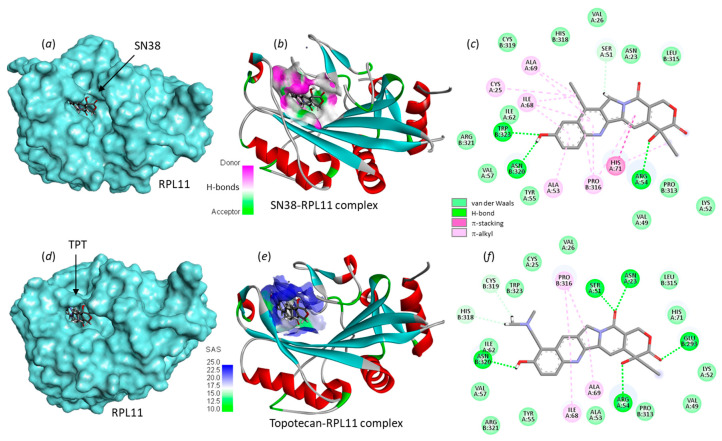
Molecular models of SN38 and topotecan bound to ribosomal protein L11 (RPL11). The upper part (**a**–**c**) shows SN38 bound to site Cys25, with (**a**) the molecule inserted into the protein cavity and (**b**) a detailed view of the SN38 binding site with the H-bond donor/acceptor groups colored (color code indicated). (**c**) Binding map contacts for SN38 bound to RPL11. The lower part (**d**–**f**) shows TPT bound to site Cys25, (**d**,**e**) a detailed view of the binding area and the solvent-accessible surface (SAS). (**f**) Binding map contacts for TPT bound to RPL11 (color code indicated).

In this case, the CAST analysis revealed a single binding site, located around residue Cys25. The different camptothecin derivatives were docked to this site, so as to determine the binding energies (Table 3). The results are similar to those obtained with RPL15. The best binder is SN38, and the weakest binder is CPT. Molecular models of SN38 and TPT bound to RPL11 are shown in Figure 7. In both cases, the drug sits into a large open cavity. The small molecule is not deeply inserted into a protein hole, as in the case of RPL15 at site Phe129. Here the drug has more freedom to move inside the cavity, but tightly interacts with the protein using both ends of the molecule, the lactone portion in contact with Arg54 and the 10-OH group on the A-ring in contact with Asn320 (Figure 7). The four drugs rank in the order SN38 > BLT > TPT > CPT (more negative ΔE values). In this case, the ΔE value measured with SN38 is extremely favorable (ΔE = −112.7 kcal/mol) and comparable to the value measured upon binding of the drug to the topoisomerase I-DNA complex. The calculated ΔE value is slightly less negative with belotecan and significantly less negative with topotecan. The docking analysis strongly suggests that RPL11 could represent a target for camptothecin-based products, at least for SN38, which seems to be particularly well adapted for binding to the Cys25 site. The analysis provides encouraging results to study experimentally the interaction of camptothecin derivatives with ribosomal proteins and the cellular consequences of the protein–drug interactions.

## 3. Discussion

The effects of camptothecins on nucleic acid and protein synthesis have been known since the early 1970s. CPT itself has been shown to block ribosome formation [34], and later the effect was linked to the specific capacity of the natural product to inhibit topoisomerase I, stabilizing the topoisomerase I-DNA covalent complex with single-stranded DNA breaks [35]. For about 50 years, camptothecins have been used as tools to manipulate topoisomerase I in cells and living organisms, and different anticancer drugs have been designed and approved based on the camptothecin scaffold [36,37]. However, beyond topoisomerase I, a few other protein targets have been advanced for these camptothecins. The 60S ribosomal protein RPL15 is one of the most recent target proteins proposed for topotecan (TPT). The drug has been reported recently to stabilize RNA G-quadruplex (RG4), so as to downregulate RG4-containing host protein factors implicated in SARS-CoV-2 infection [38]. At the ribosomal level, Yamada and coworkers have demonstrated that binding of TPT to RPL15 inhibited preribosomal subunit formation, and notably the interaction between RLP15 and RPL4. The drug-binding process induces a DAMP-mediated antitumor immune activation independent of topoisomerase I [26]. Our computational analysis of the interaction between RPL15 and TPT indicates that the interaction is entirely plausible and probably not restricted to TPT but also valid for SN38, the main metabolite of the anticancer drug irinotecan, which is largely used to treat advanced solid tumors [1]. We have located the potential binding sites for the camptothecins within the structure of RPL15, and provided structural information to define the drug-binding process. There are at least three key elements implicated in the interaction: (i) the lactone E-ring and the pendant 20-OH group both essential to the stability of the drug–protein complexes; (ii) the planar aromatic system, which allows stacking interactions with aromatic amino acids (Tyr59, His71), and (iii) the 10-OH group on the A-ring of TPT and SN38 often involved in H-bonding interaction with the protein. In contrast, the C-9 alkyl side chain, which distinguishes SN38, TPT and BLT, is not a prime element for binding to the ribosomal protein. These structural elements are important to comprehend the binding process and to help design new compounds.

RPL15 has been shown to interact with over 10 other proteins during the assembly of the 50S ribosomal structure [39]. It is a small subunit (15 kDa) of the ribosomal complex but an essential component for the maintenance of the nucleolar structure and formation of pre-60S subunits in nucleoli. RPL15 is involved in human colon carcinogenesis and is viewed as a potential target for colon cancer therapy [40]. In fact, the protein is dysregulated in various types of cancers, being notably downregulated in pancreatic ductal adenocarcinoma [41] but frequently upregulated in liver cancer (hepatocellular carcinoma, HCC) and gastric cancer [42]. The overexpression of RPL15 in gastric cancer is associated with tumor cell proliferation [43]. In gastric cancer cells, the interaction of RPL15 with the interferon-inducible protein p56 contributes to cell growth regulation. In this context, siRNA-targeting RPL15 was shown to reduce the growth rate of gastric cancer cells [44]. In HCC, RPL15 was shown to play crucial roles in tumor progression and metastasis, and as such, it is considered a promising candidate for targeted therapies [42]. The protein is also implicated in colon carcinogenesis [40], and recently, the expression of the ribosomal protein gene *RPL15* was found to be significantly upregulated in metastatic triple-negative breast cancer cells [45]. There exists also a related mitochondrial ribosomal protein L15 (MRPL15) whose abnormal expression is related to tumorigenesis [46,47]. It is therefore important to identify potential small molecule ligands and effectors for this ribosomal protein.

Small molecules capable of regulating the expression and/or function of RPL15 have been rarely described. In fact, there are only two examples. The first one refers to the pan-inhibitor of Aurora kinases danusertib, which has been shown to repress RPL15 signaling, notably negatively regulating the AURKB/p70S6K/RPL15 axis, and the effect leads to cell death (by apoptosis and autophagy) of human leukemia cells [48]. It is an indirect effect, but it confirms the interest in targeting RPL15. The second example is that of TPT with direct binding and regulation of RPL15 [26]. Camptothecins apparently represent a unique series of compounds usable as templates for the design of RPL15 modulators. The chemistry of camptothecins is extremely well known; there are hundreds of CPT analogs and derivatives, which are so many products that could be exploited to search for RPL15 inhibitors. Our docking analysis provides initial elements to identify RPL15 binding compounds in the camptothecin series.

The case of RPL11 is also interesting because this ribosomal protein has been shown previously to modulate the sensitivity of cancer cells to various topoisomerase inhibitors, including TPT [29]. RPL11 is a regulator of p53 stability, and DNA damage induced by topoisomerase (I or II) inhibitors alters the nucleolus vs. nucleoplasm location of RPL11 and subsequently the activity of p53 [49,50]. Through this process, topoisomerase inhibitors can alter the RPL11-MDM2-p53 signaling pathway [51]. However, our analysis suggests that there may also be a more direct means to interfere with RPL11, through drug binding to RPL11, notably in the case of SN38 particularly well adapted for binding to the Cys25 site. It can be a topoisomerase I-independent process to regulate the functioning of RPL11.

A few anticancer small molecules have been shown to induce nucleolar stress with a specific implication of the p53/RPL11-Mdm2 pathway, such as the NEDD8 inhibitor MLN4924 [52], mTOR inhibitors such as temsirolimus [53], and the kinase inhibitor olaparib [54]. This is the case also for the acridine derivative CID-765471, which can activate p53 through the RPL11/HDM2 pathway (without causing DNA damage) and induces nucleolar disruption [55]. Whether these products directly target RPL11 or not is not known at present, but there are good reasons to consider the protein as a valid anticancer target. Recently, RPL11 mimetics have been designed to target MDM-2 and a compound (S9) that potently binds to MDM2 was identified as a potent anticancer agent [56]. The compound was designed based on the crystal structure of the interface between RPL11 and MDM2. Another option can be to target RPL11, for example using molecules designed on the SN38 scaffold. Our work opens novel perspectives to the design of RPL11 regulatory molecules. There may be novel options to modulate the assembly of ribosomal proteins, in particular RPL15 and RPL11, with camptothecin-based molecules. The impaired ribosome biogenesis checkpoint is viewed as a target for the development of new anticancer therapies [57]. In this sense, molecules like SN38 and TPT may represent novel regulators of this ribosomal checkpoint.

A final cautionary note is important. The present work is a computational analysis intended to raise hypotheses and to propose novel directions for subsequent drug design and experimental binding studies. This in silico investigation was performed using validated methods and based on past experience and expertise learned through multiple rigorous studies with other drug–target systems [58,59,60]. We are well informed of the merits of the method, but also the limits of application [61,62]. Computer-aided drug discovery (CADD) is a useful approach, but experimental validation (wet-lab experiments) of the in silico data will be essential [63].

## 4. Materials and Methods

### 4.1. Molecular Structures and Software

The three-dimensional structure of the 80S human ribosome, which includes protein RPL15, was retrieved from the Protein Data Bank www.rcsb.org (accessed on 20 January 2023) under the PDB code 4UG0. It is a high-resolution structure (2.9–3.6 Å resolution) obtained by cryo-electron microscopy and atomic model building [30]. The structure of ribosomal protein L11 derives from the high-resolution (2.40 Å) X-ray structure of the human MDM2-RPL11 complex (PDB: 4XXB) [32]. Docking experiments were performed using the GOLD software (GOLD 5.3 release, Cambridge Crystallographic Data Centre, Cambridge, UK). Molecular graphics and analyses were performed using Discovery Studio Visualizer, Biovia 2020 (Dassault Systèmes BIOVIA Discovery Studio Visualizer 2020; San Diego, CA, USA, Dassault Systèmes, 2020). Potential drug-binding sites for the different molecules were searched using the web server Computed Atlas of Surface Topography of proteins (CASTp) 3.0 and visualized with the molecular modeling software Chimera v1.15 https://www.cgl.ucsf.edu/chimera/ (accessed on 20 January 2023) [31].

### 4.2. In Silico Molecular Docking Procedure

The process used includes the following steps:

(1) Monte Carlo (MC) conformational search of the ligand using the BOSS (Biochemical and Organic Simulation System) software v4.9 http://zarbi.chem.yale.edu/software.html (accessed on 20 January 2023), freely available to academic users. The structure of the ligand was optimized using a classical MC conformational search procedure, as described in BOSS [64]. A conformational analysis has been performed to define the best starting geometries for each compound. Energy minimization was carried out to identify all minimum-energy conformers, leading to the identification of a unique conformer for the free ligand. Within BOSS, MC simulations were performed in the constant-temperature and constant-pressure ensemble (NPT).
(2) Evaluation of the free energy of hydration for the chosen structure of the ligand. The molecular mechanics/generalized Born surface area (MM/GBSA) procedure was used to evaluate the free energies of hydration (ΔG) [65]. MC search and computation of ΔG were performed within BOSS using the xMCGB script according to procedures given in references [65,66]. The best ligand structure was then used in the docking procedure.
(3) Definition of the ribosomal protein–ligand sites of interaction. Drug-binding sites were searched using CASTp 3.0, a convenient tool for active site prediction. With the 4XXB (RPL11) structure, based on shape complementarity criteria, the flexible amino acids are Asn23, Cys25, Ser51, Arg54, Ile68, His71, Ser317, His318, Asn320 and Trp323. With the 4UG0 (RPL15) structure, the flexible amino acids are (i) Lys54, Lys56, Glu57, Tyr59, Ile135, Asp136, His139, Ile142, Thr148, and Trp150 (site Ile135) and (ii) Trp11, Leu23, Arg26, Gln29, Tyr30, Leu33, His37, Thr43, Arg63, Phe129 (site Phe129). Shape complementarity and geometry considerations favor a docking grid centered in the volume defined by the central amino acid. Within the binding site, the side chains of the specific amino acids were considered fully flexible during docking.
(4) Docking procedure using GOLD. In our typical docking process, 100 energetically reasonable poses (according to the ChemPLP scoring function) are retained while searching for the correct binding mode of the ligand. The decision to maintain a trial pose is based on ranked poses, using the PLP fitness scoring function (which is the default in GOLD version 5.3 used here) [67]. Six poses are kept. The empirical potential energy of the interaction ΔE for the ranked complexes was evaluated using the simple expression ΔE(interaction) = E(complex) − [E(protein) + E(ligand)]. Calculations of the final energy are performed on the basis of the SPASIBA spectroscopic force field. The corresponding parameters are derived from vibrational wavenumbers obtained in the infrared and Raman spectra of a large series of compounds including organic molecules, amino acids, saccharides, nucleic acids and lipids.
(5) Validation using the SPASIBA force field. This last step is considered essential to define the best protein–ligand structure. The spectroscopic SPASIBA (Spectroscopic Potential Algorithm for Simulating Biomolecular conformational Adaptability) force field has been specifically developed to provide refined empirical molecular mechanics force field parameters [68]. SPASIBA empirical energies of interaction are calculated as described [69,70]. SPASIBA (integrated into CHARMM) [71] has been shown to be excellent at reproducing crystal-phase infrared data. The same procedure was used to establish molecular models for the various drug–protein complexes.

## 5. Conclusions

Based on the recent discovery that the anticancer drug topotecan (TPT) can target the 60S ribosomal protein RPL15 as a means to inhibit preribosomal subunit formation and to induce an antitumor immune activation [26], we have identified the potential binding site for TPT and camptothecin derivatives on RPL15. Two potential sites emerge from our molecular docking analysis, located around residues Phe129 and Ile135. Compound SN38, the active metabolite of the anticancer drug irinotecan, can bind well to each site, but binding to the larger site Phe129 is apparently preferred. Its RPL15 binding capacity is superior to that of TPT and belotecan (BLT). SN38 may also bind to the analogous protein RPL11. The two ribosomal proteins RPL11 and RPL15 offer binding sites for camptothecin derivatives. Structure–binding relationships have been delineated. They could guide the design of small molecules targeting RPL15 and/or RPL11, both considered antitumor targets. The study also shed light on the mechanism of action of topotecan beyond its primary capacity to interfere with topoisomerase I.

## Figures and Tables

**Table 1:** Calculated potential energy of interaction (ΔE) and free energy of hydration (ΔG) for the interaction of the camptothecins with RPL15.

Compounds	ΔE (kcal/mol)	ΔG (kcal/mol)	ΔE (kcal/mol)	ΔG (kcal/mol)
*Site*	*Site Phe129*		*Site Ile135*	
Belotecan	−63.30	−15.70	−80.40	−11.90
Camptothecin	−51.90	−15.50	−65.10	−19.20
SN38	−83.70	−16.00	−79.30	−14.50
Topotecan	−65.90	−16.70	−66.30	−11.30

**Table 2:** Calculated potential energy of interaction (ΔE) for the binding of the camptothecins to different molecular targets.

Compounds	Target	PDB	ΔE (kcal/mol)
SN38	RPL15	4UGO	−83.7 *
Camptothecin	TopoI-DNA complex	1TI8	−114.20
Topotecan	TopoI-DNA complex	1K4T	−80.10
Topotecan	ABCG2 transporter	7NEZ	−67.55

* Data for site Phe129 (details in Table 1).

**Table 3:** Calculated potential energy of interaction (ΔE) and free energy of hydration (ΔG) for the interaction of the camptothecins with RPL11.

Compounds	ΔE (kcal/mol)	ΔG (kcal/mol)
SN38	−112.70	−20.30
Belotecan	−93.50	−30.40
Topotecan	−80.80	−21.20
Camptothecin	−67.55	−23.20

## Data Availability

Data sharing not applicable.

## References

[1] Bailly C. (2019). Irinotecan: 25 years of cancer treatment. Pharmacol. Res..

[2] Bailly C., Thuru X., Quesnel B. (2020). Combined cytotoxic chemotherapy and immunotherapy of cancer: Modern times. NAR Cancer.

[3] Pommier Y., Nussenzweig A., Takeda S., Austin C. (2022). Human topoisomerases and their roles in genome stability and organization. Nat. Rev. Mol. Cell Biol..

[4] Tariq S., Kim S.Y., Monteiro de Oliveira Novaes J., Cheng H. (2021). Update 2021: Management of Small Cell Lung Cancer. Lung.

[5] Petrelli F., Ghidini A., Luciani A. (2021). Topotecan or other agents as second-line therapy for relapsed small-cell lung cancer: A meta-analysis of randomized studies. Mol. Clin. Oncol..

[6] Frampton J.E. (2020). Liposomal Irinotecan: A Review in Metastatic Pancreatic Adenocarcinoma. Drugs.

[7] Milano G., Innocenti F., Minami H. (2022). Liposomal irinotecan (Onivyde): Exemplifying the benefits of nanotherapeutic drugs. Cancer Sci..

[8] Goldenberg D.M., Sharkey R.M. (2019). Antibody-drug conjugates targeting TROP-2 and incorporating SN-38: A case study of anti-TROP-2 sacituzumab govitecan. MAbs.

[9] Bardia A., Hurvitz S.A., Tolaney S.M., Loirat D., Punie K., Oliveira M., Brufsky A., Sardesai S.D., Kalinsky K., Zelnak A.B. (2021). ASCENT Clinical Trial Investigators. Sacituzumab Govitecan in Metastatic Triple-Negative Breast Cancer. N. Engl. J. Med..

[10] Fontes M.S., Vargas Pivato de Almeida D., Cavalin C., Tagawa S.T. (2022). Targeted Therapy for Locally Advanced or Metastatic Urothelial Cancer (mUC): Therapeutic Potential of Sacituzumab Govitecan. OncoTargets Ther..

[11] Staker B.L., Hjerrild K., Feese M.D., Behnke C.A., Burgin A.B., Stewart L. (2002). The mechanism of topoisomerase I poisoning by a camptothecin analog. Proc. Natl. Acad. Sci. USA.

[12] Talukdar A., Kundu B., Sarkar D., Goon S., Mondal M.A. (2022). Topoisomerase I inhibitors: Challenges, progress and the road ahead. Eur. J. Med. Chem..

[13] Martín-Encinas E., Selas A., Palacios F., Alonso C. (2022). The design and discovery of topoisomerase I inhibitors as anticancer therapies. Expert. Opin. Drug Discov..

[14] Kowal J., Ni D., Jackson S.M., Manolaridis I., Stahlberg H., Locher K.P. (2021). Structural Basis of Drug Recognition by the Multidrug Transporter ABCG2. J. Mol. Biol..

[15] Rasouli A., Yu Q., Dehghani-Ghahnaviyeh S., Wen P.C., Kowal J., Locher K.P., Tajkhorshid E. (2023). Differential dynamics and direct interaction of bound ligands with lipids in multidrug transporter ABCG2. Proc. Natl. Acad. Sci. USA.

[16] Haque A., Baig G.A., Alshawli A.S., Sait K.H.W., Hafeez B.B., Tripathi M.K., Alghamdi B.S., Mohammed Ali H.S.H., Rasool M. (2022). Interaction Analysis of MRP1 with Anticancer Drugs Used in Ovarian Cancer: In Silico Approach. Life.

[17] Wong D.V.T., Holanda R.B.F., Cajadov A.G., Bandeira A.M., Pereira J.F.B., Amorim J.O., Torres C.S., Ferreira L.M.M., Lopes M.H.S., Oliveira R.T.G. (2021). TLR4 deficiency upregulates TLR9 expression and enhances irinotecan-related intestinal mucositis and late-onset diarrhoea. Br. J. Pharmacol..

[18] Wong D.V.T., Ribeiro-Filho H.V., Wanderley C.W.S., Leite C.A.V.G., Lima J.B., Assef A.N.B., Cajado A.G., Batista G.L.P., González R.H., Silva K.O. (2019). SN-38, the active metabolite of irinotecan, inhibits the acute inflammatory response by targeting toll-like receptor 4. Cancer Chemother. Pharmacol..

[19] Tam J.S.Y., Pei J.V., Coller J.K., Prestidge C.A., Bowen J.M. (2022). Structural insight and analysis of TLR4 interactions with IAXO-102, TAK-242 and SN-38: An in silico approach. In Silico Pharmacol..

[20] Gasimli K., Raab M., Becker S., Sanhaji M., Strebhardt K. (2022). The Role of DAPK1 in the Cell Cycle Regulation of Cervical Cancer Cells and in Response to Topotecan. J. Cancer.

[21] Khageh Hosseini S., Kolterer S., Steiner M., von Manstein V., Gerlach K., Trojan J., Waidmann O., Zeuzem S., Schulze J.O., Hahn S. (2017). Camptothecin and its analog SN-38, the active metabolite of irinotecan, inhibit binding of the transcriptional regulator and oncoprotein FUBP1 to its DNA target sequence FUSE. Biochem. Pharmacol..

[22] Hoang V.T., Verma D., Godavarthy P.S., Llavona P., Steiner M., Gerlach K., Michels B.E., Bohnenberger H., Wachter A., Oellerich T. (2019). The transcriptional regulator FUBP1 influences disease outcome in murine and human myeloid leukemia. Leukemia.

[23] Lee B., Min J.A., Nashed A., Lee S.O., Yoo J.C., Chi S.W., Yi G.S. (2019). A novel mechanism of irinotecan targeting MDM2 and Bcl-xL. Biochem. Biophys. Res. Commun..

[24] Zhang J.L., Sharma P.L., Li C.J., Dezube B.J., Pardee A.B., Crumpacker C.S. (1997). Topotecan inhibits human immunodeficiency virus type 1 infection through a topoisomerase-independent mechanism in a cell line with altered topoisomerase I. Antimicrob. Agents Chemother..

[25] Zeng X., Zhu S., Lu W., Liu Z., Huang J., Zhou Y., Fang J., Huang Y., Guo H., Li L. (2020). Target identification among known drugs by deep learning from heterogeneous networks. Chem. Sci..

[26] Yamada S., Kitai Y., Tadokoro T., Takahashi R., Shoji H., Maemoto T., Ishiura M., Muromoto R., Kashiwakura J.I., Ishii K.J. (2022). Identification of RPL15 60S Ribosomal Protein as a Novel Topotecan Target Protein That Correlates with DAMP Secretion and Antitumor Immune Activation. J. Immunol..

[27] Kitai Y., Kawasaki T., Sueyoshi T., Kobiyama K., Ishii K.J., Zou J., Akira S., Matsuda T., Kawai T. (2017). DNA-Containing Exosomes Derived from Cancer Cells Treated with Topotecan Activate a STING-Dependent Pathway and Reinforce Antitumor Immunity. J. Immunol..

[28] Kitai Y. (2022). Elucidation of the Mechanism of Topotecan-induced Antitumor Immune Activation. Yakugaku Zasshi.

[29] Ishihara Y., Nakamura K., Nakagawa S., Okamoto Y., Yamamoto M., Furukawa T., Kawahara K. (2022). Nucleolar Stress Response via Ribosomal Protein L11 Regulates Topoisomerase Inhibitor Sensitivity of P53-Intact Cancers. Int. J. Mol. Sci..

[30] Khatter H., Myasnikov A.G., Natchiar S.K., Klaholz B.P. (2015). Structure of the human 80S ribosome. Nature.

[31] Tian W., Chen C., Lei X., Zhao J., Liang J. (2018). CASTp 3.0: Computed atlas of surface topography of proteins. Nucleic Acids Res..

[32] Connolly M.L. (1983). Solvent-accessible surfaces of proteins and nucleic acids. Science.

[33] Zheng J., Lang Y., Zhang Q., Cui D., Sun H., Jiang L., Chen Z., Zhang R., Gao Y., Tian W. (2015). Structure of human MDM2 complexed with RPL11 reveals the molecular basis of p53 activation. Genes Dev..

[34] Wu R.S., Kumar A., Warner J.R. (1971). Ribosome formation is blocked by camptothecin, a reversible inhibitor of RNA synthesis. Proc. Natl. Acad. Sci. USA.

[35] Hsiang Y.H., Hertzberg R., Hecht S., Liu L.F. (1985). Camptothecin induces protein-linked DNA breaks via mammalian DNA topoisomerase I. J. Biol. Chem..

[36] Pommier Y. (2009). DNA topoisomerase I inhibitors: Chemistry, biology, and interfacial inhibition. Chem. Rev..

[37] Thomas A., Pommier Y. (2019). Targeting Topoisomerase I in the Era of Precision Medicine. Clin. Cancer Res..

[38] Tong Q., Liu G., Sang X., Zhu X., Fu X., Dou C., Jian Y., Zhang J., Zou S., Zhang G. (2023). Targeting RNA G-quadruplex with repurposed drugs blocks SARS-CoV-2 entry. PLoS Pathog..

[39] Lieberman K.R., Noller H.F. (1998). Ribosomal protein L15 as a probe of 50 S ribosomal subunit structure. J. Mol. Biol..

[40] Dong Z., Jiang H., Liang S., Wang Y., Jiang W., Zhu C. (2019). Ribosomal Protein L15 is involved in Colon Carcinogenesis. Int. J. Med. Sci..

[41] Yan T.T., Fu X.L., Li J., Bian Y.N., Liu D.J., Hua R., Ren L.L., Li C.T., Sun Y.W., Chen H.Y. (2015). Downregulation of RPL15 may predict poor survival and associate with tumor progression in pancreatic ductal adenocarcinoma. Oncotarget.

[42] Shi R., Liu Z. (2022). RPL15 promotes hepatocellular carcinoma progression via regulation of RPs-MDM2-p53 signaling pathway. Cancer Cell Int..

[43] Wang H., Zhao L.N., Li K.Z., Ling R., Li X.J., Wang L. (2006). Overexpression of ribosomal protein L15 is associated with cell proliferation in gastric cancer. BMC Cancer.

[44] Hsu Y.A., Lin H.J., Sheu J.J., Shieh F.K., Chen S.Y., Lai C.H., Tsai F.J., Wan L., Chen B.H. (2011). A novel interaction between interferon-inducible protein p56 and ribosomal protein L15 in gastric cancer cells. DNA Cell Biol..

[45] Zhao W., Li X., Nian W., Wang J., Wang X., Sun L., Zhu Y., Tong Z. (2021). Ribosome Proteins Represented by RPL27A Mark the Development and Metastasis of Triple-Negative Breast Cancer in Mouse and Human. Front. Cell Dev. Biol..

[46] Zeng Y., Shi Y., Xu L., Zeng Y., Cui X., Wang Y., Yang N., Zhou F., Zhou Y. (2021). Prognostic Value and Related Regulatory Networks of MRPL15 in Non-Small-Cell Lung Cancer. Front. Oncol..

[47] Xu H., Zou R., Li F., Liu J., Luan N., Wang S., Zhu L. (2021). MRPL15 is a novel prognostic biomarker and therapeutic target for epithelial ovarian cancer. Cancer Med..

[48] He S.J., Shu L.P., Zhou Z.W., Yang T., Duan W., Zhang X., He Z.X., Zhou S.F. (2016). Inhibition of Aurora kinases induces apoptosis and autophagy via AURKB/p70S6K/RPL15 axis in human leukemia cells. Cancer Lett..

[49] Wang H., Feng J., Zhou T., Wei L., Zhou J. (2018). Involvement of RPL11 in the enhancement of P53 stability by a podophyllum derivative, a topoisomerase II inhibitor. Cell Biol. Int..

[50] Rao Z., Shen J., Wang J., Zhang Z., Zhou J., Zhu J., Chen J., Chen W., Wang H. (2022). The role of PICT1 in RPL11/Mdm2/p53 pathway-regulated inhibition of cell growth induced by topoisomerase IIα inhibitor against cervical cancer cell line. Biochem. Pharmacol..

[51] Franklin D.A., Liu S., Jin A., Cui P., Guo Z., Arend K.C., Moorman N.J., He S., Wang G.G., Wan Y.Y. (2022). Ribosomal protein RPL11 haploinsufficiency causes anemia in mice via activation of the RP-MDM2-p53 pathway. J. Biol. Chem..

[52] Bailly A., Perrin A., Bou Malhab L.J., Pion E., Larance M., Nagala M., Smith P., O’Donohue M.F., Gleizes P.E., Zomerdijk J. (2016). The NEDD8 inhibitor MLN4924 increases the size of the nucleolus and activates p53 through the ribosomal-Mdm2 pathway. Oncogene.

[53] Goudarzi K.M., Nistér M., Lindström M.S. (2014). mTOR inhibitors blunt the p53 response to nucleolar stress by regulating RPL11 and MDM2 levels. Cancer Biol. Ther..

[54] Han T., Tong J., Wang M., Gan Y., Gao B., Chen J., Liu Y., Hao Q., Zhou X. (2022). Olaparib Induces RPL5/RPL11-Dependent p53 Activation via Nucleolar Stress. Front. Oncol..

[55] Morgado-Palacin L., Llanos S., Urbano-Cuadrado M., Blanco-Aparicio C., Megias D., Pastor J., Serrano M. (2014). Non-genotoxic activation of p53 through the RPL11-dependent ribosomal stress pathway. Carcinogenesis.

[56] Wang B., Gao J., Zhao Z., Zhong X., Cui H., Hou H., Zhang Y., Zheng J., Di J., Liu Y. (2022). Identification of a small-molecule RPL11 mimetic that inhibits tumor growth by targeting MDM2-p53 pathway. Mol. Med..

[57] Bursać S., Prodan Y., Pullen N., Bartek J., Volarević S. (2021). Dysregulated Ribosome Biogenesis Reveals Therapeutic Liabilities in Cancer. Trends Cancer.

[58] Bailly C., Vergoten G. (2022). Binding of Vialinin A and *p*-Terphenyl Derivatives to Ubiquitin-Specific Protease 4 (USP4): A Molecular Docking Study. Molecules.

[59] Vergoten G., Bailly C. (2022). Molecular docking study of britannin binding to PD-L1 and related anticancer pseudoguaianolide sesquiterpene lactones. J. Recept. Signal Transduct. Res..

[60] Vergoten G., Bailly C. (2022). Molecular docking study of GSK-3β interaction with nomilin, kihadanin B, and related limonoids and triterpenes with a furyl-δ-lactone core. J. Biochem. Mol. Toxicol..

[61] Sharma P.P., Bansal M., Sethi A., Poonam Pena L., Goel V.K., Grishina M., Chaturvedi S., Kumar D., Rathi B. (2021). Computational methods directed towards drug repurposing for COVID-19: Advantages and limitations. RSC Adv..

[62] Fukunishi Y., Higo J., Kasahara K. (2022). Computer simulation of molecular recognition in biomolecular system: From in silico screening to generalized ensembles. Biophys. Rev..

[63] Gentile F., Oprea T.I., Tropsha A., Cherkasov A. (2023). Surely you are joking, Mr Docking!. Chem. Soc. Rev..

[64] Jorgensen W.L., Tirado-Rives J. (1996). Monte Carlo versus Molecular Dynamics for conformational sampling. J. Phys. Chem..

[65] Jorgensen W.L., Tirado-Rives J. (2005). Molecular modeling of organic and biomolecular systems using BOSS and MCPRO. J. Comput. Chem..

[66] Jorgensen W.L., Ulmschneider J.P., Tirado-Rives J. (2004). Free energies of hydration from a generalized Born model and an ALL-atom force field. J. Phys. Chem. B.

[67] Jones G., Willett P., Glen R.C., Leach A.R., Taylor R. (1997). Development and validation of a genetic algorithm for flexible docking. J. Mol. Biol..

[68] Meziane-Tani M., Lagant P., Semmoud A., Vergoten G. (2006). The SPASIBA force field for chondroitin sulfate: Vibrational analysis of D-glucuronic and N-acetyl-D-galactosamine 4-sulfate sodium salts. J. Phys. Chem. A.

[69] Vergoten G., Mazur I., Lagant P., Michalski J.C., Zanetta J.P. (2003). The SPASIBA force field as an essential tool for studying the structure and dynamics of saccharides. Biochimie.

[70] Lagant P., Nolde D., Stote R., Vergoten G., Karplus M. (2004). Increasing Normal Modes Analysis Accuracy: The SPASIBA Spectroscopic Force Field Introduced into the CHARMM Program. J. Phys. Chem. A.

[71] Homans S.W. (1990). A molecular mechanical force field for the conformational analysis of oligosaccharides: Comparison of theoretical and crystal structures of Man alpha 1-3Man beta 1-4GlcNAc. Biochemistry.

